# Successful BRCA2 Mutation Detection via Tissue-Based Gene Panel Testing in Post-bone Marrow Transplant Breast Cancer: Implications for Precision Medicine

**DOI:** 10.7759/cureus.78729

**Published:** 2025-02-08

**Authors:** Chisato Takeuchi, Seiichi Imanishi, Namiko Tanaka, Nobuyoshi Kittaka

**Affiliations:** 1 Department of Breast Surgery, Osaka Rosai Hospital, Osaka, JPN

**Keywords:** bone marrow transplantation, bracanalysis, brca1/2 mutations, parp inhibitors, tissue-based gene panel testing

## Abstract

*BRCA1* and *BRCA2* (*BRCA1/2*) mutation screening is recommended for patients with advanced breast cancer or early breast cancer suspected to have hereditary origins based on family history or tumor characteristics. Blood-based testing is the standard approach, but it is not feasible in patients who have undergone bone marrow transplantation due to the replacement of blood cells with donor-derived cells.

We report a case of a patient who, following bone marrow transplantation, required an alternative to blood-based germline *BRCA1/2* screening. A germline *BRCA2* mutation was inferred through tissue-based gene panel testing on a tumor sample, using variant allele frequency analysis. This approach allowed the initiation of targeted therapy with poly(ADP-ribose) polymerase (PARP) inhibitors, resulting in sustained progression-free intervals.

This case demonstrates the potential of tissue-based gene panel testing to identify actionable germline mutations in breast cancer patients when blood-based testing is not feasible, thereby expanding therapeutic options in complex clinical scenarios.

## Introduction

Breast cancer has the highest incidence rate among cancers affecting women, with approximately 5% to 10% of cases classified as hereditary [1-3]. Among these hereditary cases, nearly half are attributed to pathogenic variants in the *BRCA1 *and *BRCA2 *(*BRCA1/2*) genes in the germline [4]. Germline *BRCA1/2* screening is conventionally performed using blood samples, with BRACAnalysis testing being the standard diagnostic method for detecting these mutations. BRACAnalysis CDx is a diagnostic kit that uses quantitative polymerase chain reaction (PCR) and bi-directional Sanger sequence analysis to detect and classify variants in the *BRCA1 *and *BRCA2 *genes. Recently, it has become possible to detect *BRCA1/2* mutations using next-generation sequencing panels (NGS) technology [5].

However, BRACAnalysis is not recommended for individuals who have undergone bone marrow transplantation. This is due to the chimerism resulting from the replacement of hematopoietic cells with donor-derived cells, which can mask the patient’s genetic profile, leading to inaccurate assessments in genetic testing [6]. This limitation underscores the challenges involved in accurately identifying germline *BRCA *mutations in post-transplant patients.

When pathogenic *BRCA *mutations are detected, there is an increased risk of contralateral breast cancer and ovarian cancer [7,8]. Specifically, for *BRCA1 *mutation carriers, the cumulative risk of ovarian cancer by age 80 is 44% (95% CI, 36%-53%), and the cumulative risk of contralateral breast cancer 20 years after a breast cancer diagnosis is 40% (95% CI, 35%-45%). Similarly, for *BRCA2 *mutation carriers, the cumulative risk of ovarian cancer by age 80 is 17% (95% CI, 11%-25%), and the cumulative risk of contralateral breast cancer 20 years after a breast cancer diagnosis is 26% (95% CI, 20%-33%) [9].

Consequently, the National Comprehensive Cancer Network (NCCN) guidelines (Version 2.2025) recommend risk-reducing measures, such as contralateral mastectomy or salpingo-oophorectomy, for carriers of these mutations [10]. These interventions have been shown to significantly reduce the risk of developing contralateral breast cancer and ovarian cancer. For example, risk-reducing salpingo-oophorectomy can reduce the risk of ovarian cancer by approximately 80%, and contralateral risk-reducing mastectomy significantly reduces the risk of breast cancer, with a relative risk of 0.07 (95% CI 0.04-0.15) [10-11]. The NCCN guidelines recommend that risk-reducing salpingo-oophorectomy typically be offered to *BRCA1 *mutation carriers between the ages of 35 and 40, and to *BRCA2 *mutation carriers between the ages of 40 and 45, taking into account individual risk factors and preferences. The timing of contralateral mastectomy should also be individualized based on factors such as age, family history, and personal preferences [10].

In addition to preventive measures, poly(adenosine diphosphate-ribose) polymerase (PARP) inhibitors have emerged as a promising therapeutic option, providing significant benefits for patients with *BRCA*-mutated breast cancer [12-14]. *BRCA1/2* encode proteins critical for homologous recombination DNA repair. Cancer cells with mutations in *BRCA1/2* are deficient in the repair mechanism for DNA double-strand breaks, leaving these tumors highly dependent on the repair pathway for single-strand breaks. This pathway is regulated by the enzyme PARP; PARP inhibitors exploit the principle of synthetic lethality to selectively kill tumor cells with this deficiency [8-9]. Clinical trials have demonstrated the efficacy of PARP inhibitors in improving progression-free survival (PFS) and invasive disease-free survival in these patients. The OlympiAD trial reported a significantly longer median PFS in the olaparib group compared with the standard therapy group (7.0 months vs. 4.2 months; hazard ratio (HR) 0.58; 95% confidence interval (CI) 0.43-0.80; P < 0.001) [12]. Similarly, the OlympiA trial demonstrated a significantly higher three-year invasive disease-free survival in the olaparib group compared with the placebo group (85.9% vs 77.1%; HR 0.58; 99.5% CI 0.41-0.82; P < 0.001) [13]. Furthermore, the EMBRACA trial showed a longer median PFS in the talazoparib group compared with the standard treatment group (8.6 months vs. 5.6 months; HR 0.54; 95% CI 0.41-0.71; P < 0.001) [14].

Here, we report a metastatic breast cancer case where a germline *BRCA2 *mutation was identified via tissue-based gene panel testing, necessitated by prior bone marrow transplantation precluding standard blood-based *BRCA1/2* testing. This approach successfully identified a germline *BRCA1/2* mutation in a post-transplant patient, addressing the diagnostic challenge in this population. This underscores the feasibility and utility of comprehensive genetic testing when blood-based testing is infeasible.

## Case presentation

A 41-year-old woman presented with a complex medical history of breast cancer and subsequent treatments. She was initially diagnosed with left breast cancer at the age of 29. The tumor was hormone receptor-positive and HER2-negative. She underwent a mastectomy with sentinel lymph node biopsy, followed by postoperative endocrine therapy. At age 31, she was diagnosed with contralateral (right) breast cancer. She underwent partial resection and axillary dissection, followed by endocrine therapy and radiotherapy. Tumor characteristics were as follows: estrogen receptor (ER) 90%, progesterone receptor (PR) 5%, HER2 1+, and Ki67 index 20%.

At age 33, the patient underwent a bone marrow transplant for aplastic anemia. Two years later, at age 35, a left-sided pleural effusion was detected and diagnosed as a malignant pleural effusion, suggestive of breast cancer recurrence. Weekly paclitaxel therapy achieved a clinical complete response (CR). Clinical CR was determined by maintaining a progression-free period for three years with monthly tumor marker (carcinoembryonic antigen (CEA), cancer antigen 15-3 (CA15-3)) testing and imaging evaluations with US and CT every three months. At age 37, she experienced a recurrence in the right breast along with right-sided pleural effusion and underwent a right mastectomy. Pathology of the surgical specimen revealed ER >10%, PR >10%, HER2 1+, histological grade 2, and Ki67 index 30%.

**Figure 1:**
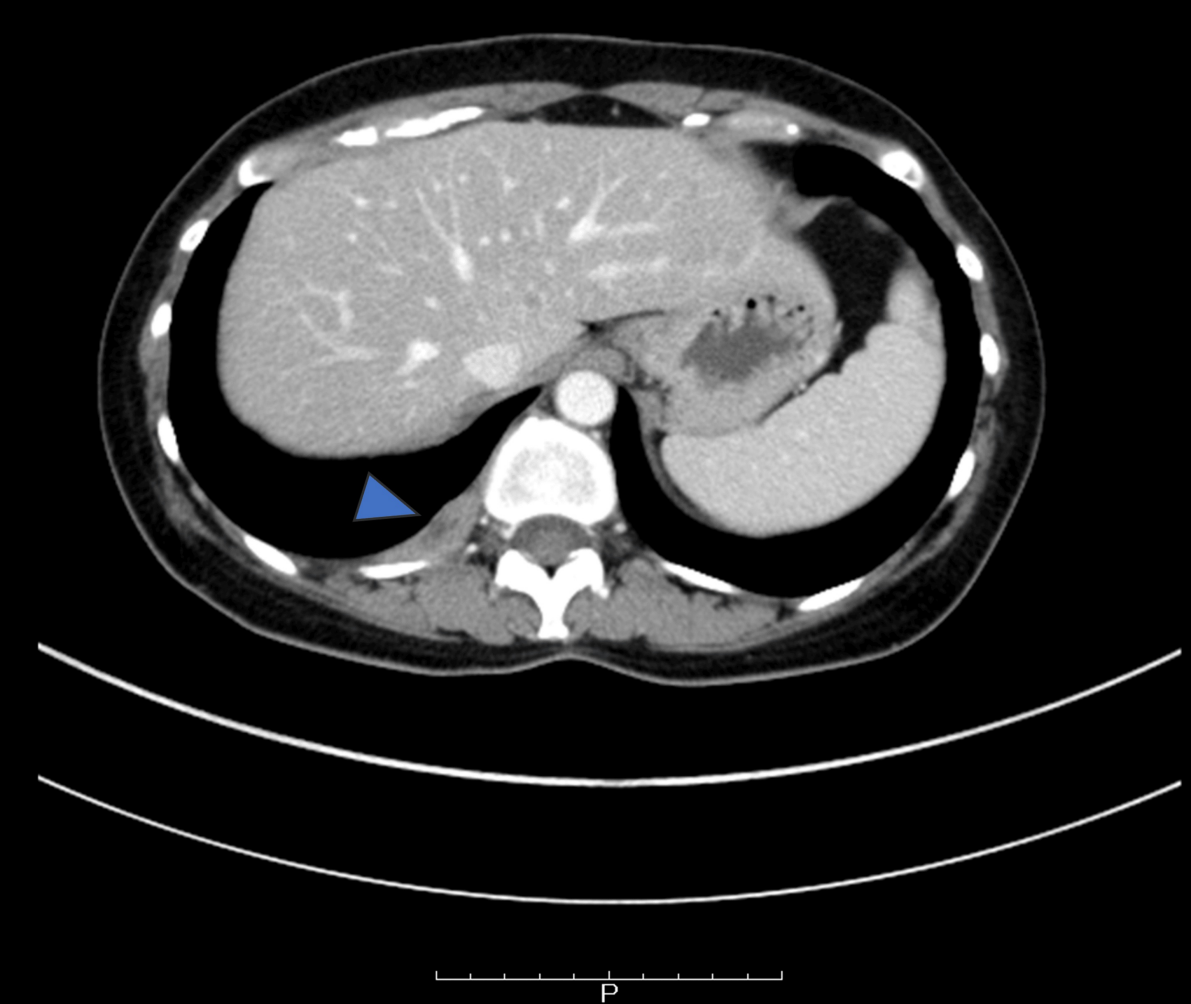
CT image at the time of transfer to our hospital showing malignant pleural effusion (indicated by arrow).

**Figure 2:**
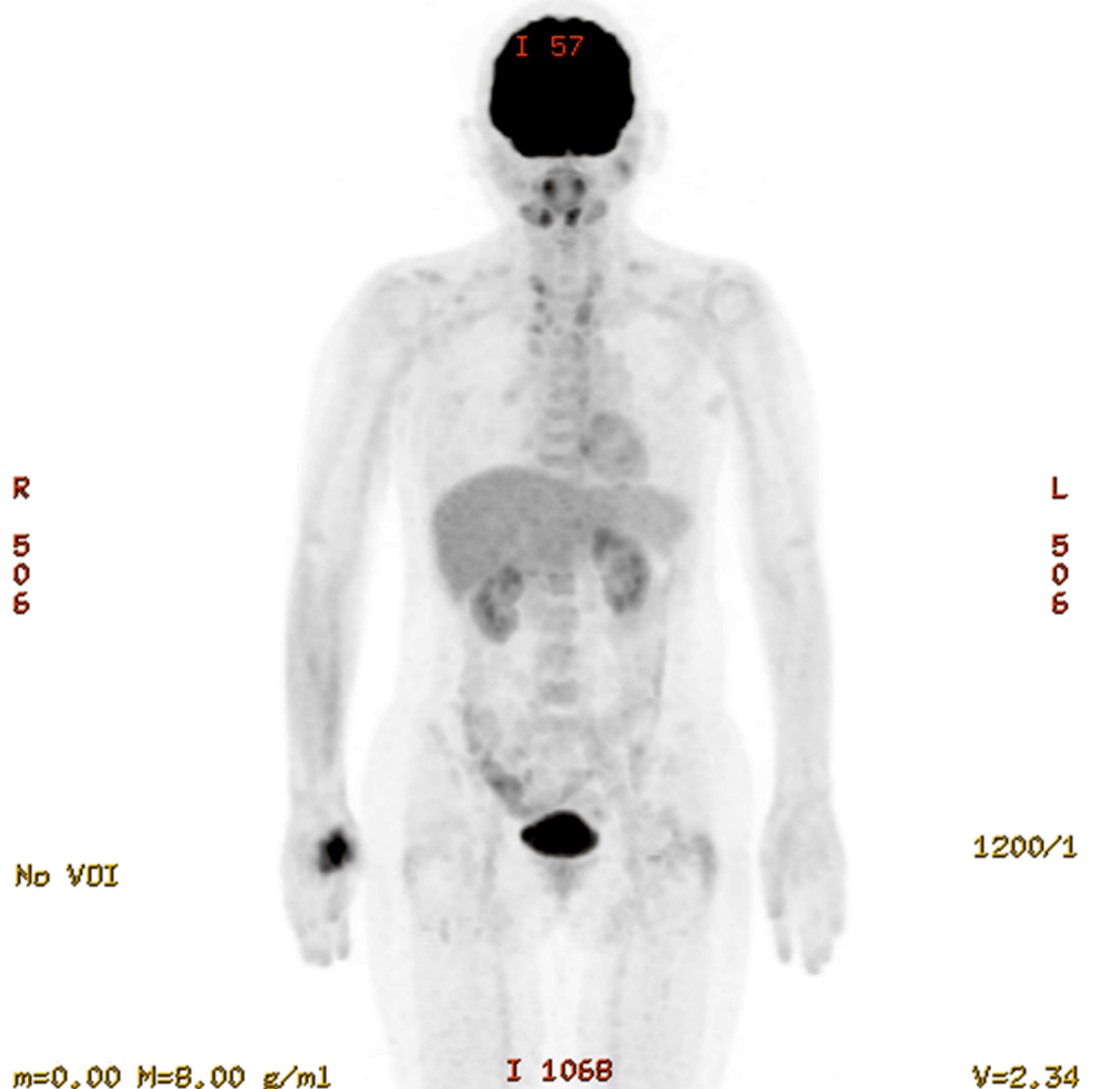
The 18F-fluorodeoxyglucose positron emission tomography (18F-FDG PET/CT) scan revealed no significant uptake indicative of recurrence. This is an 18F-fluorodeoxyglucose positron emission tomography (18F-FDG PET/CT) scan after administration of cyclophosphamide (FEC) and docetaxel (DTX). No significant uptake was observed, which would indicate recurrence.

Following the surgery, the patient was referred to our hospital for systemic therapy. At this time, malignant pleural effusion persisted (Figure 1). Given the patient's previous bone marrow transplant history and the presence of pleural effusion, anastrozole was selected for its favorable pharmacokinetic and safety profile. Initially, the addition of abemaciclib was supported by MONARCH-3 trial data, though it required discontinuation due to toxicity [15]. Given the patient's previous tamoxifen failure, we initiated sequential fluorouracil, epirubicin, and cyclophosphamide (FEC)-docetaxel (DTX) chemotherapy. The regimen was optimized with four cycles each of FEC and DTX to balance efficacy with cardiotoxicity risk. A clinical CR was confirmed by an 18F-fluorodeoxyglucose positron emission tomography (18F-FDG PET/CT) scan (Figure 2).

Although the patient had no family history of cancer, her early-onset, bilateral breast cancers raised the suspicion of hereditary breast and ovarian cancer syndrome (HBOC). Tumor-based testing was chosen over circulating tumor DNA analysis due to the patient's prior bone marrow transplantation. Therefore, tumor-based gene panel testing (FoundationOne®, Foundation Medicine Inc., Cambridge, MA, USA) was performed to evaluate the suitability of PARP inhibitors, which identified a pathogenic *BRCA2* mutation (R2318*) with a variant allele frequency (VAF) of 71.52%. The sample had 50% cellularity, and no copy number alterations in *BRCA2* were detected (Table 1).

**Table 1:** Genomic findings from the FoundationOne® CDx gene panel test *BRCA2* mutation was identified and variant allele frequency was highly efficient. Aberrant cell fraction; cancer content in the sample; variant allele frequency; proportion of cells with mutations PARP: poly(ADP-ribose) polymerase

Parameter	Value
Tumor cellularity	50%
Mutation identified	*BRCA2* R2318*
Mutation classification	Nonsense mutation
Variant allele frequency	71.25%
Disease-related genes	*ERBB2* (negative), *BRCA1*(negative)
Microsatellite status	Stable
Tumor mutational burden	3.78 mutations/Mb
Clinical interpretation	Eligible for PARP inhibitors

Based on these findings, experts meeting at Osaka University Hospital recommended treatment with the PARP inhibitor olaparib. Olaparib was administered for 12 months, with ongoing treatment. The patient has maintained a complete response for 36 months.

## Discussion

In breast cancer treatment, the detection of *BRCA1/2* gene mutations is critically important, as it guides both therapeutic decisions and genetic counseling. Traditionally, genetic testing for *BRCA *mutations has relied on blood samples, which are the most practical and widely available method, although alternative sources such as saliva are increasingly being utilized in certain cases. Although research into alternative sources like saliva and hair is ongoing, these methods are not yet implemented in clinical practice [16,17]. When blood-based genetic testing is not feasible, as demonstrated in this case, tissue-based genetic testing offers a valuable alternative.

For patients who have undergone bone marrow transplantation, such as the patient in the present case, blood-derived cells are replaced with donor-derived cells. Consequently, the patient’s own germline genetic information cannot be directly analyzed through blood testing, posing challenges for conventional BRACAnalysis. In such scenarios, gene panel testing using tissue samples has the significant advantage of enabling the analysis of both tumor and normal tissues when matched samples are available, providing a viable alternative to blood-derived samples.

In this case, the tissue sample contained both cancerous and normal cells, with 50% of the tissue being cancerous. Given the absence of copy number changes, if the cancer content is 50% and only somatic mutations are present, the VAF would not be expected to exceed 50%. Therefore, combining cancer content and VAF can provide a reliable estimate of germline mutations. This methodology was particularly beneficial for identifying the *BRCA2 *germline mutation (R2318*) in this patient.

While reports of administering PARP inhibitors based on gene panel testing are emerging, there is a lack of detailed analyses on inferring germline mutations from tissue samples. This case provides valuable insight into the practical application of tissue-based testing for germline mutation identification, particularly in patients with unique constraints such as a history of bone marrow transplantation. It is often unclear whether such mutations were confirmed as germline in these studies [18]. The present case underscores the potential utility of tissue-based gene panel testing, such as FoundationOne®, in identifying actionable mutations and guiding treatment with PARP inhibitors, particularly when blood-based testing is not feasible and high-quality tissue samples are available. For patients in whom traditional germline testing methods are challenging, this approach offers a promising alternative for detecting mutations and tailoring personalized therapies, though its reliability depends on careful sample selection and interpretation. Ideally, confirmation of a germline mutation should be performed using a specimen unaffected by the tumor, such as an oral mucosal or skin biopsy. However, in this case, such a confirmatory test was not performed, which remains a limitation.

Beyond therapeutic issues, confirmation of germline mutations has clinical importance beyond treatment, as it impacts hereditary cancer risk assessment, surveillance strategies, and genetic counseling for families. Given these implications, patients with suspected germline *BRCA *mutations should be encouraged to undergo genetic counseling for comprehensive risk assessment and preventive measures. This case highlights both the clinical value of tissue-based gene panel testing and the importance of follow-up evaluation to address hereditary cancer risk.

## Conclusions

The detection of *BRCA1/2* germline mutations plays a critical role in guiding breast cancer management, particularly in patients with suspected hereditary risks or complex clinical histories. When blood-based testing is not feasible, such as after bone marrow transplantation, tissue-based gene panel testing provides a promising alternative for identifying actionable mutations, though careful interpretation is essential. In certain scenarios, tissue-based testing can infer germline *BRCA1/2* mutations, which may enable personalized treatment strategies. However, this approach requires careful analysis to distinguish germline mutations from somatic alterations, as the lack of a matched normal sample can make definitive classification challenging.

This case demonstrates the clinical utility of tissue-based testing in guiding PARP inhibitor therapy for a patient with a history of bone marrow transplantation. It highlights the potential of this approach to expand therapeutic options in similar complex clinical scenarios. While further research is warranted to validate the broader applicability of tissue-based testing for germline mutation detection, this case highlights its potential as a valuable tool in clinical settings where conventional germline testing is not feasible. Future studies should focus on refining methodologies and establishing standardized guidelines for its use.

However, tissue-based testing also has its limitations. For example, it is difficult to clearly distinguish germline and somatic mutations without comparison with normal tissue. Furthermore, the proportion of tumor cells, the accuracy of testing techniques, and other factors may influence the interpretation of results. Future research should develop methods to more accurately distinguish between germline and somatic mutations, improve analysis techniques, and establish methods. Furthermore, the method needs to be standardized by conducting large-scale studies and accumulating case numbers. Despite these limitations, this approach has the potential to expand genetic testing options and contribute to the development of personalized medicine.
